# Combination therapy with silibinin, pegylated interferon and ribavirin in a patient with hepatitis C virus genotype 3 reinfection after liver transplantation: a case report

**DOI:** 10.1186/1752-1947-8-257

**Published:** 2014-07-21

**Authors:** Johanna Knapstein, Marcus A Wörns, Peter R Galle, Tim Zimmermann

**Affiliations:** 1 I. Department of Internal Medicine, Johannes Gutenberg-University, Langenbeckstraße 1, 55131 Mainz, Germany

**Keywords:** Antiviral therapy, Liver transplantation, Silibinin

## Abstract

**Introduction:**

Hepatitis C virus reinfection occurs universally after liver transplantation with accelerated cirrhosis rates of up to 30% within 5 years after liver transplantation. Management of hepatitis C virus reinfection is complicated by drug interactions and pre-treatment. Dual antiviral therapy with pegylated interferon and ribavirin only reaches sustained virological response rates of approximately 30% after liver transplantation. With the approval of the viral NS3/4A protease and NS5B ribonucleic acid -dependent ribonucleic acid polymerase inhibitors, combination therapy offers new therapeutic options resulting in considerably higher sustained virological response rates in the non-transplant setting. However, silibinin has also shown potent antiviral activity in non-responders to dual therapy.

**Case presentation:**

We report the first case of antiviral therapy with pegylated interferon and ribavirin in combination with silibinin post-liver transplantation in a 50-year-old Caucasian man with genotype 3 reinfection with prior non-response.

Silibinin was administered at a dose of 20mg/kg/day intravenously for 2 weeks and continued orally for 47 weeks in combination with a 48-week pegylated interferon and ribavirin therapy (180μg/week and 800mg/day), which was started on day 8. Pegylated interferon and ribavirin doses were adapted to 135μg/week and 600mg/day. After 4 weeks of therapy, the viral load declined 6 log_10_ and became undetectable in week 6, resulting in a sustained virological response 24 weeks after the end of therapy.

In general, antiviral therapy was well tolerated. Side effects included pruritus and anaemia leading to erythropoietin therapy.

**Conclusions:**

Combination therapy with pegylated interferon, ribavirin and silibinin resulted in sustained virological response 24 weeks after the end of therapy in a patient reinfected with hepatitis C virus genotype 3 who was a prior non-responder after liver transplantation. Silibinin therapy may offer a new therapeutic option for patients reinfected with non-genotype 1 hepatitis C virus who have had a liver transplanted and are non-responders.

## Introduction

Hepatitis C virus (HCV) reinfection occurs universally after liver transplantation (LT) [1]. Under immunosuppression, the time course of recurrent HCV is accelerated, with cirrhosis rates of up to 30% within 5 years of LT [2]. So far, antiviral therapy has been limited to a combination of pegylated interferon (peg-IFN) and ribavirin (RBV) with sustained virological response (SVR) rates of approximately 30% post-LT [3-5]. With the approval of the viral NS3/4A protease inhibitor (PI) simeprevir and NS5B ribonucleic acid (RNA)-dependent RNA polymerase inhibitor (RdRpI) sofosbuvir, combination therapy offers new therapeutic options resulting in considerably higher SVR rates of 66% and 75% in treatment-naïve patients infected with HCV genotype 1 in the non-transplant setting [6,7]. However, the management of HCV reinfection after LT is complicated by drug interactions, tolerability and pre-treatment [8,9]. Therefore an individual treatment regimen is often required. However, PI-based triple therapy is limited to patients infected with HCV genotype 1. To date, very few data exist on the treatment of recurrent non-genotype 1 HCV-infection after LT.

As silibinin has been shown to be a potent antiviral agent in prior non-responders to dual therapy [10,11], we present here the first case of antiviral therapy with peg-IFN and RBV in combination with silibinin post-LT in a genotype 3 reinfected patient with prior non-response.

## Case presentation

**Figure 1:**
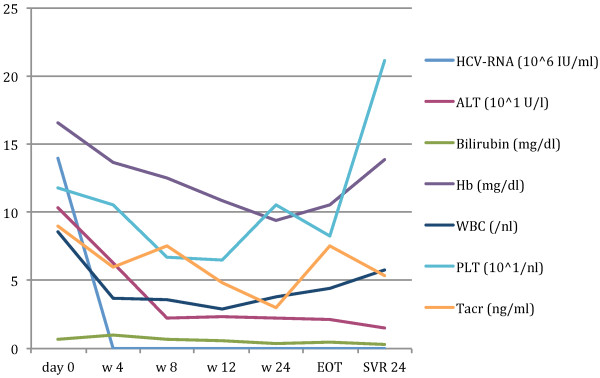
**Course of hepatitis C virus-ribonucleic acid (10**^**6 **^**IU/mL), alanine aminotransferase (10**^**1**^**U/L), haemoglobin (mg/dL), white blood cells (cells/nL) and platelets (10**^**1**^**/nL) during therapy.** After 4 weeks of therapy, viral load declined 6 log_10_ and became negative in week 6 resulting in SVR 24. Side effects included pruritus and anaemia, leading to the application of erythropoietin. Abbreviations: ALT, alanine aminotransferase; EOT, end-of-treatment; Hb, haemoglobin; HCV-RNA, hepatitis C virus-ribonucleic acid; PLT, platelets; SVR, sustained virological response; Tacr, tacrolimus; w, week; WBC, white blood cells.

A 50-year-old Caucasian man underwent a liver transplant in 2008 due to hepatocellular carcinoma based on chronic HCV genotype 3a-associated liver cirrhosis. HCV was diagnosed in 1996. He was pre-treated with dual antiviral therapy pre-LT in 1997, resulting in primary non-response. In 2008, recurrent HCV was detected in liver biopsy. Therefore, he underwent a second attempt of dual antiviral therapy over 48 weeks, resulting in a relapse. He was referred to our LT out-patient clinic in a good state of general health (body mass index 26.2, 180cm, 85kg) with a request for antiviral therapy. HCV-RNA measured 14×10^6^ IU/mL (COBAS® TaqMan® HCV test, version 2.0, Roche Diagnostics AG, Rotkreuz, Switzerland; lower limit of quantification: 25IU/mL; lower limit of detection: 10IU/mL). His serum transaminases were slightly elevated: alanine aminotransferase 103U/L, upper limit of normal (ULN <50IU/L; aspartate aminotransferase 114U/L, ULN 35IU/L). All other liver values were within the normal range (alkaline phosphatase, gamma-glutamyltransferase, bilirubin, international normalized ratio and albumin). Haemoglobin, leukocyte and thrombocyte counts were 16.6mg/dL, 8.6/nL and 118/nL, respectively (Figure 1). Side diagnoses included hypertension and diabetes mellitus type 2.

As silibinin has shown potent antiviral activity in prior non-responders to dual therapy, informed consent was given for a triple combination therapy of silibinin, peg-IFN and RBV. He was given tacrolimus 1mg twice a day (BID) and mycophenolate-mofetil 500mg BID for immunosuppression as well as co-medication with amlodipine (5mg/day) and pantoprazole (40mg/day); insulin injections were also continued. Silibinin was administered 20mg/kg/day intravenously for 2 weeks and continued orally 560mg/day for 47 weeks in combination with a 48-week peg-IFN and RBV therapy with 180μg/week and 800mg/day started on day 8. Peg-IFN and RBV doses were adapted to 135μg/week and 600mg/day. After 4 weeks of therapy, the viral load declined 6 log_10_ and became undetectable in week 6, resulting in SVR 24. Therapy was well tolerated; side effects included dyspnoea, pruritus and anaemia, leading to the application of 250mg BID ursodeoxycholic acid and 30μg erythropoietin/week. Red blood cell, leukocyte and platelet counts, as well as tacrolimus through levels, were checked once a week.

## Discussion

Antiviral therapy of recurrent HCV after LT is an issue of high interest and the time course of fibrosis progression is accelerated under immunosuppression [2]. Improvements in antiviral therapy are urgently required as SVR rates for dual therapy in patients who are HCV positive after LT are low [3,4]. PI and RdRpI-based combination for antiviral therapy in non-LT cohorts leads to significantly higher SVR rates compared to a dual therapy [6-10]. However, the management of HCV post-LT is complicated by drug interactions, side effects and pre-treatment [11,12].

However, PI-based triple therapy is limited to patients infected with HCV genotype 1. To date, very few data exist on the therapy of recurrent non-genotype 1 HCV-infection after LT.

*In vitro* experiments have shown that HCV replication is significantly inhibited by silibinin [13]. Silibinin has a strong anti-oxidative effect [14]. This may improve the response to IFN in non-responders to dual therapy since oxidative stress leads to impaired IFN signalling [15]. However, evidence for beneficial effects in humans has been equivocal. Therefore, the efficacy and safety of high-dose intravenous silibinin, followed by a lower dose oral application in combination with dual antiviral therapy, were evaluated by Ferenci *et al*. [16] in 2008 in chronic HCV genotype 1, 2 and 4 infected former non-responders. This study showed that silibinin is well tolerated and reveals a substantial antiviral effect against HCV in non-responders [16]. A recent study reported similar results in the transplant setting for patients reinfected with HCV genotype 1 [17]. We present here the first case of successful antiviral treatment with silibinin in combination with peg-IFN and RBV in a patient reinfected with HCV genotype 3 after LT with prior non-response to dual therapy. Treatment with silibinin might result in an IFN-sensitising effect in IFN non-responders following LT.

## Conclusions

Combination therapy of peg-IFN, RBV and silibinin resulted in SVR 24 in a patient who had a liver transplanted, who was reinfected with HCV genotype 3 and who was therapy naïve. Combination therapy with silibinin might be a useful approach for the therapy of recurrent non-genotype 1 HCV infection after LT.

## Consent

Written informed consent was obtained from the patient for publication of this case report and the accompanying images. A copy of the written consent is available for review by the Editor-in-Chief of this journal.

## Abbreviations

BID: Twice a day; HCV: Hepatitis C virus; LT: Liver transplantation; peg-IFN: Pegylated interferon; PI: Protease inhibitor; RBV: Ribavirin; RdRpI: RNA-dependent RNA polymerase inhibitor; RNA: Ribonucleic acid; SVR: Sustained virological response; ULN: Upper limit of normal.

## Competing interests

The authors declare that they have no competing interests.

## Authors’ contributions

JK collected the data and drafted the manuscript. PRG, MAW and TZ conceived of the case, and participated in therapy design and coordination and helped to draft the manuscript. All authors read and approved the final manuscript.
